# Supplementary material for: Evaluation of serverless computing for scalable execution of a joint variant calling workflow
Source: PLoS One. 2021 Jul 9;16(7):e0254363. doi: 10.1371/journal.pone.0254363 (PMC8270184; doi:10.1371/journal.pone.0254363)
Supplement: Attachment — Submitted filename: Respone-SWEEP -Genomics Manuscript.pdf [file pone.0254363.s002.pdf]

Dear Editors,

Thank you for your insightful comments on our submitted manuscript, “Evaluation of serverless computing for scalable execution of a joint variant calling workflow.” We have reviewed the comments and added clarifications to the manuscript as needed. Please see our responses (in **bold**) to each reviewer comment (in *italics*) below.

After adding these proposed changes, we believe this paper is now suitable to be published in PLOS One. Thank you again for your consideration.

Sincerely,

Aji John
Kristiina Ausmees
Kathleen Muenzen

Reviewer 1 Comments:

1. Authors need to highlight the difference from the previously published work (John et al. "SWEEP: Accelerating Scientific Research Through Scalable Serverless Workflows"). The original work that introduces SWEEP also presents a case study (Section 4.1) which focuses on modeling the variant calling workflow. From the workflow modeling perspective, the workflow presented in this paper is not significantly different from the already published case study. It would be helpful if authors emphasize how the workflow presented in this paper is different.

The workflow presented in the previous study was entirely scalable, whereas the current workflow presents scaling challenges that would realistically be faced by people using SWEEP. This clarification was added in lines 44-46 in the manuscript.

2. Additionally, since no source code was referenced in the previously-published work, for readers not from the bioinformatics field it might look like a small delta from the previously-published work.

The past work was on a single chromosome vs now we are performing joint variant calling on all autosomes to test a more realistic workload. This clarification was added in lines 43-46.

3. Both in the paper and in the GitHub repository authors state that the modeled workflow was executed on AWS and Azure. However, in the Discussion section (page 12, line 222) authors mention that due to technical limitations it was not possible to execute some tasks hosted on AWS. It is not quite clear, which tasks were executed on which provider and how this influenced the modeled workflow(s). It would be helpful if authors emphasize which actions are required from users to model-deploy-execute a SWEEP-based workflow, and how AWS/Azure come into play here. In case some tasks from the modeled workflow were executed ONLY on Azure, this means that the actual workflow is not a single-cloud workflow and, among other issues, requires considering costs of inter-cloud data transfers too.

Workflows tasks were executed either on AWS or Azure; for the purpose of simplicity - simple routing was wired into the workflow CaaS task definitions. In the GVCF workflow the SWEEP configuration (on the backend) was pointed to Azure for CaaS, and AWS for FaaS tasks. SWEEP offers the users task level overrides to a specific CP or system level routing where the optimum destination of a task (regardless of type) is chosen by SWEEP. Paragraph (Lines 237-239) amended to elaborate the routing mechanisms. The product documentation (docs.sweep.run) has been augmented to emphasize user actions.

4. The implementation requirements related to function/container code are not clear. Since AWS and Azure impose different requirements on source code, integration with service offerings, and packaging formats, is it the case that SWEEP workflows can only be enacted on multiple providers if the code does not use provider-specific features at all? In addition, the invocation of FaaS functions can happen differently on AWS and Azure, e.g., HTTP-based using API Gateways, events, direct calls from the source code. The question is which requirements SWEEP workflows impose on the function code. In case API Gateways are used to invoke functions, the costs of API Gateway offerings have to be included into the picture as well, similar to data transfer costs if cross-cloud interactions were needed for enacting the workflow.

SWEEP provides a way to deploy the functions to various CP(s) and has the knowledge of the resource namespace. SWEEP has adapters built-in to invoke FaaS or CaaS artifacts across different CP(s); adapters are aware of the individual CP(s) API specifications. Lines 82-84 address the clarification.

5. It is stated that SWEEP workflows require actual functions/containers to be already deployed on the target provider, although its API has endpoints for uploading functions. As stated before, it would be helpful if authors clarify the actions required from users to model/deploy (both workflow and functions/containers), and execute a SWEEP workflow.

A task is considered registered when the task is deployed via SWEEP to a particular CP. SWEEP offers APIs to deploy the task, however, it's in a limited beta stage. Lines 79-80 address the clarification.

6. Authors need to explain why using SWEEP is more beneficial than using provider-specific orchestrators such as AWS Step Functions(SF) and Azure Durable Functions(DF). Both SF and DF are mature orchestrators supporting using FaaS/CaaS offerings from AWS and Azure which provide out-of-the-box service integration, error handling, etc. From the description of the process it is not clear, why SWEEP is more beneficial to use. Firstly, constructs allowing implementing composite integration patterns such as scatter-gather are present in SF and DF, together with means to automate the deployment of code/workflows, whereas this work has to be done manually in SWEEP. Moreover, since it is not quite clear which communication type is used to trigger functions/containers, the costs of using SWEEP might incur not only the per-request costs, but also additional service offerings such as AWS API Gateway, making the overall costs more expensive than using SF, for example. In the best case, authors might provide a comparison between SWEEP and solutions from AWS/Azure to highlight pluses/minuses of using SWEEP workflows, e.g., cross-cloud workflow orchestration becomes possible, bioinformatics pipelines execution is more straightforward, etc.

The paragraph containing the motivation for using SWEEP has been extended to stress the fact that SWEEP supports multiple clouds, and that the main downside of cloud provider specific orchestrators is the associated vendor lock-in. Lines 27-29 and 36-37 address this clarification.

7. Phrasing:

-- page 3, line 35: "As SWEEP is fully built on the serverless framework, ..."

This is confusing, Serverless framework is a deployment automation tool, whereas SWEEP is presented as a serverless orchestrator / workflow management system.

The phrase "serverless framework" here did not refer to the particular deployment automation tool with this name, but the general execution model. The phrase has been changed to "execution model". Added the clarification in lines 30-42.

8. Phrasing:

-- page 4, line 64: "... using the Function-as-a-Service (FaaS) paradigm."

-- page 4, line 66: "... using Container-as-a-Service (CaaS) services."

"cloud service model" instead of paradigm/services?

Both of the above have been changed to "execution model" as well. Lines 75-77 address this clarification.

Reviewer 2 Comments:

1. The domain for applying the workflows is genomics, in particular genome sequencing with joint-variant workflows. It is not the first article discussing the combination of serverless computing and bioinformatics. In 2019, Niu et al. studied high-performance sequence comparison, and Crespo-Cepeda et al. investigated the potential of the combination in general. Unfortunately, neither of these works are referenced so it remains unclear how much overlap and novelty there is.

We are specifically looking at the combination of serverless computing, bioinformatics and workflow management systems (WMS) in this paper. While we demonstrated that serverless computing + WMS can help overcome some of the challenges of executing large-scale genomics workflows, we also showed that serverless computing has some major drawbacks for non-scalable components of workflows. Previous publications have focused more on the benefits of serverless computing in bioinformatics, rather than the drawbacks. This clarification was added in lines 45-48 on the manuscript.

2. SWEEP uses functions and containers to execute workflow tasks. It is not clear if CaaS refers to traditional long-running container services (e.g. pure Docker or Kubernetes), or to short-lived executions (e.g. Fargate, Google Cloud Run, Knative), and whether the statelessness is hence an issue for both. Fargate and ACI are mentioned later on, yet it is not clear why all tasks per runtime get the same memory allocation, and why the memory allocated to functions is much smaller (e.g. 3 GB instances would be possible with Lambda and would speed up execution). That should all be clarified in the section on definition of workflows. More technical details on the "as opposed to Docker Hub base images" would also be useful - whether it merely concerns the installation of additional packages, or also the interface to the containers and how information is passed to them and results are retrieved. It looks like in total (Fig. 1) 5 out of 18 workflow steps are cloud functions, whereas much the paper makes it sound as if most of the workflow was. What were the decisions to go for either technology in each step? This knowledge would greatly contribute to the value of the manuscript as it would help readers facing similar challenges.

We used the same runtime configuration for all the tasks to achieve simplicity. However, the feedback is keen, we hope to address optimum runtime configuration for tasks in future releases of SWEEP. Lines 104 - 107 clarifies the division of tasks types in the GVCF workflow, and the motivation behind it.

3. Fig. 2 positions a cost of zero to a runtime of around 3 hours. First, the graph starts at around 15000s, or around 4 hours; and furthermore, it would imply 0 or negative cost for shorter runtimes whereas the pricing model of FaaS/CaaS is linear except for free tiers. It is not clear if the graph was created and interpreted correctly. Fig. 2 is also not referenced from the text. Some attention should be given to it because it represents the value proposition of the paper, that using SWEEP would be cost-effective.

We were not implying that at zero there was a cost incurred; however, we see the source of confusion as the number of samples start at 2. We have clarified the same in Figure 2 caption. Lines 198 and 209 refers to Fig 2.

4. The manuscript as a whole could be made more readable. The acronym GVCF is not explained; presumably the G stands for Gathering but the rest is unclear. What does it do and why did you choose it as representative workflow that would allow to generalise findings?

GVCF is an enhanced version of the VCF file format--a "genomic" VCF. The "GVCF workflow" is an eponymously named workflow that uses the GVCF file format as an intermediate to parallelize joint variant calling as much as possible. It is commonly used in the field of genomics to call variants on entire cohorts and contains both scalable and less scalable components. This clarification was added in lines 58-61 of the manuscript.

5. A typical JSON excerpt of GVCF in your own SWEEP language would further help in understanding the nature of the workflow and how it would map to cloud functions or containers. Moreover, most providers - including AWS - offer their own languages for serverless workflows; a short statement of differentiation would also be helpful (that might focus on portability and ability to also include containerised endpoints).

We have uploaded the SWEEP workflow definition to our GitHub repository - https://github.com/SWEEP-Inc/GVCF

6. Moreover, most providers - including AWS - offer their own languages for serverless workflows; a short statement of differentiation would also be helpful (that might focus on portability and ability to also include containerised endpoints).

The paragraph containing the motivation for using SWEEP has been extended to stress the fact that SWEEP supports multiple clouds, and that the main downside of cloud provider specific orchestrators is the associated vendor lock-in. Clarification was added in lines 32-42.

7. Nitpicks:

Fig. 1 has dashed edges but the caption talks of dotted edges.

This has been addressed in the main text and legend for Figure 1.

8. For transparency reasons, it would be helpful to point out in the review materials that SWEEP is linked to a startup. KM is listed on the SWEEP website as lead software engineer but the competing interests only mention KA and AJ.

KM was shortly employed by DotMote Labs at the time of developing the joint-variant calling workflows; we have amended the verbiage in the competing interest's section of our cover letter. Website has been changed as well as KM is not currently employed with SWEEP.
