# Supplementary material for: Evaluation of serverless computing for scalable execution of a joint variant calling workflow
Source: PLoS One. 2021 Jul 9;16(7):e0254363. doi: 10.1371/journal.pone.0254363 (PMC8270184; doi:10.1371/journal.pone.0254363)
Supplement: Attachment — Submitted filename: Respone-SWEEP -Genomics Manuscript.pdf [file pone.0254363.s003.pdf]

Dear Editors,

Thank you for your insightful comments on our submitted manuscript, “Evaluation of serverless computing for scalable execution of a joint variant calling workflow.” We have reviewed the recent comments and added clarifications to the manuscript and to our documentation as needed. Please see our responses (in **bold**) to each reviewer comment (in *italics*) below.

After adding these proposed changes, we believe this paper is now suitable to be published in PLOS One. Thank you again for your consideration.

Sincerely,

Aji John
Kristiina Ausmees
Kathleen Muenzen

Reviewer 1 Comments:

In this work, the authors presented the modeling and execution of GATK best-practice joint variant calling pipeline using SWEEP, a process engine offered as a service, which supports executing tasks hosted on AWS and Azure.

The revision addresses issues raised by reviewers related to the original submission.

The difference between the previous work and the workflow presented in this work is explained.

Authors also provide clarifications on how cloud provider services were used, highlighting that this workflow can only be executed in a multi-cloud setting due to limitations of certain cloud services.

Authors provide a high-level clarification on interaction with provider-hosted function/containers, which is probably motivated by SWEEP commercialization plans. While the aspects of interaction with provider-specific services are not directly required in the context of this work, such technicalities influence the code implementation requirements. In particular, to achieve the vendor-agnosticism mentioned as one of the main motivations for using SWEEP, functions/containerized applications might have to be developed without relying on provider-specific libraries. For example, if an Azure-specific Java annotations are used in a function, deploying it to AWS Lambda would require additional efforts (or does the system handle such cases for users?). The documentation also does not specify how functions/containerized applications need to be implemented, e.g., implementing certain interfaces, adhering to certain data formats. In my opinion, highlighting such requirements would strengthen the work.

Overall, I am glad to accept this work when more details on code implementation requirements are provided.

The discussion regarding provider-specific libraries and how this affects the use of SWEEP is an interesting one, but one that we feel is out of scope for this paper. The main goal of the study at hand is to discuss the use of SWEEP to implement this particular workflow, and not a description of the framework itself. We have therefore tried to keep details about SWEEP to the minimum that is required for understanding the implementation and focused on the variant calling application. However, this comment is very pertinent to aspirations of any workflow provider, and certainly does apply to SWEEP. We have since incorporated some of the suggestions in our documentation on SWEEP website regarding implementation details for function/container tasks, and plan to continuously improve it by providing more examples.

Reviewer 2 Comments:

This is a revised manuscript that addresses most of the previously raised concerns. Indeed, the motivation for using SWEEP given the characteristics of the bioinformatics/genomics workflow needs is now more clear, and the authors contribute a critical view on using serverless platforms for the workflow steps not requiring these execution features. The nature of the work has not changed, it remains an experience report discussing and experimentally measuring the SWEEP migration of a given pipeline. Such experiences help the serverless systems community to achieve more applicable system designs. I have no major points to complain, but if another round of minor edits is possible, there are some more rough edges that could improve with more attention.

A critical point is the limitation on number of tasks. If each task stays within the specified disk limit and yet the task fails (and requires retries), that might be labelled more explicitly a bug in ACI, or it is some other effect that should be explained in greater detail. Given the potential blocker impact on running other large workflows, it would also be interesting to know if for instance Fargate suffers from the same effect. The preference for ACI over Fargate is explained with the greater disk size (50 vs 10 GB), but that greater disk may not be worth much when the instances fail randomly.

Adaptive selection of storage is mentioned as possible extension of the work. It would be great if some hints were given how this could be done in a way that the workflow will benefit, for instance by accomodating some latency issues by juggling between instance disk, externally mounted disk (on AWS Lambda) and managed storage (AWS S3/ABS) equivalent.

A useful addition, if space permits, would be an elaboration on the modifications of container images compared to those found on Docker Hub. Specifically, if this merely concerns the ability to invoke them in as ACI, or any other changes beyond the invocation interface.

Regarding the issues if failing tasks and the causes, a paragraph was added in order to discuss the failure of tasks in ACI. While it would be interesting to investigate the causes of the failures in more detail in the future, this is considered out of scope for the current paper. We instead aim to describe it from a user perspective, as an unexpected issue that we came across when using the services of a particular CP. Also, while previous experiments suggest that Fargate is more stable w.r.t this particular issue, we do not have data supporting this for this particular workflow and tasks, and therefore refrain from a direct comparison. We instead added a more general discussion on the stability of serverless services that a user might find relevant to consider. Please see lines 233-238 discussing these points.

For comment w.r.t to adaptive selection of storage, we note that filesystem mounting capability has since become available for functions and container-based tasks; this might be a good option to explore when disk size available for compute is limited. Lines 254-257 were added to elucidate this point.

We have also added details on SWEEP website on porting of Docker images.
